# The Cyclopædia of Practical Surgery

**Published:** 1842-01

**Authors:** 


### Art. IX.

*The Cyclopædia of Practical Surgery; including an etymological and critical terminology; detailed descriptions of instruments and apparatus; a copious bibliography; and an historical view of the progress of the science to the present day: the whole presenting a complete digest, alphabetically distributed, of the doctrines and practice of Surgery.* Conducted by W. B. Costello, assisted by numerous distinguished writers in the various departments of Medical and Surgical Science. Illustrated with numerous engravings and woodcuts. Vol. I. ABA—DYS.—*London,* 1841. Roy. 8vo, pp. 879.

The first volume of this work was completed by the publication of the eighth Part in May last. The second volume is now in progress, and, we have reason to believe, will proceed with greater speed and regularity than its predecessor. As on former occasions we have noticed the general plan of this publication, we shall proceed, at once, to give some account of the more important articles contained in the first volume now before us.

The articles in the present volume which treat of the joints and their diseases, are by Mr. Blackburn, Mr. T. S. Wells, and Dr. Little. Those on anchylosis, traumatic arthritis, and articulations, are from the pen of the first of these gentlemen, who has evidently paid much attention to diseases of the joints, and has furnished three able practical papers. Chrondritis is by Mr. Wells, and is a useful paper, affording a summary of our knowledge on a subject respecting which we are still much in want of further information. Dr. Little has contributed a supplement to the article anchylosis, in which he narrates many cases in illustration of the advantages resulting from a division of the tendons in external anchylosis. Several of the cases are instructive, but they might, we think, have been much condensed with advantage. We shall proceed to give a few extracts from some of the above papers, treating as they do of a portion of surgery which so eminently owes its advancement to the labours of English surgeons, and especially of him who now so worthily occupies the first place among them.

*Articulations.* Under this head Mr. Blackburn briefly notices the various diseases to which the joints, and especially the more complicated joints, are liable. These he divides into such as arise from *external causes*, as wounds, sprains, and luxations, and such as are due to *internal causes*. He notices in succession the several tissues which enter into the composition of joints, and alludes shortly to the diseases which affect these. In speaking of scrofulous affection of the *bone*, and of the manner in which the cartilage becomes separated in this disease, he says:

"The appearance of the remaining cartilage in this state of things is so peculiar, that it may be almost regarded as diagnostic of the disease. The removal of the cartilage commences on its attached surface, and it is not uncommon to see a flake of cartilage supported by its continuity with other portions of cartilage overhanging the diseased and excavated bone. On taking hold of this tissue it may be easily detached in flakes of considerable size from the bone beneath, which is dark, ulcerated, and denuded. In the cases of ordinary inflammation, such as are cited by Key, Mayo, and Gerdy, either granulations, by which the removal of the cartilage is supposed to be effected, are thrown out from the osseous surface, or the cellular membrane interposed between the cartilage and the bone, invisible in the healthy state, becomes highly developed and vascular; but in scrofulous cases the bone seems altogether incapable of this action, and presents, as already mentioned, a dark and denuded surface." (p. 407.)

The author notices the opinion of Mr. Key, that ulceration of the ligamentum teres is a frequent cause of disease in the hip-joint. He admits, as an unquestionable fact, that ulceration of the ligamentous tissue may take place as a primary lesion, but inclines to the opinion that, in the majority of cases, the ligament is detached by caries of the bones, rather than by a primary affection of the ligament itself. In treating of the synovial membrane, Mr. Blackburn discusses at some length the question, whether this membrane forms a shut sac, and thus lines the cartilages, or whether, as Cruveilhier, Velpeau, and others maintain, it terminates at the edges of the cartilages: he concludes in favour of the former opinion. He also maintains the doctrine of the vitality of the cartilages, as evinced both by their physiological and pathological appearances, in opposition to the opinion held by the above anatomists, that they are to be considered as mere epidermic crusts. The arguments on both these questions are stated with ability and fairness, but will scarcely admit of condensation.

*Anchylosis.* In treating of this affection Mr. Blackburn has adopted a division into external anchylosis, the result of lesions of tissues surrounding the joint; and internal, dependent on lesion of the tissues proper to the joint; a very preferable division to that usually adopted, into true and false anchylosis, depending on the bond of union being by bone or by ligamentous or other soft tissues. *External* anchylosis may originate from a variety of causes, as long-continued want of motion, diseases of cellular membrane, lesions of the muscles and tendons by paralysis, or otherwise. *Internal* anchylosis may be due to *rigidity of the ligaments.*

"Inflammation may be set up in ligamentous tissue by a sprain, a luxation, or a fracture bordering on the articulation, by gout and rheumatism. This inflammation gives rise, at first, to a swollen and softened state of the ligaments, produced by deposition of lymph between the fibres; subsequently the whole tissue becomes rigid and indurated. When in this state it has very much the character of cartilage or fibrocartilage, and no longer permits the bones to move on one another with ease and freedom....Ossification of the ligaments is assigned by some writers as a frequent cause of anchylosis, but it is certainly much rarer than is commonly supposed. The most common transformation of true ligamentous tissue is into cartilage. Internal anchylosis is sometimes produced by the formation of adhesions between the opposed surfaces of the synovial membranes. It is certain that the inflammation of this tissue does not ordinarily give rise to the effusion of plastic matter; but that it may do so occasionally is a well-established fact. The anchylosis thus formed may be firm and unyielding, but the medium of union between the articular extremities is only cellular membrane more or less condensed, while the anchylosis which ensues after the ulceration of cartilage or bone is usually effected either by bone or by ligamentous tissue." (p. 308.)

In speaking of the latter form of anchylosis succeeding to the ulceration of cartilage, Mr. Blackburn observes that

"Mr. Mayo speaks of anchylosis being produced by an effusion of false membrane from the substance of the cartilage, after an ulceration which had affected only its surface. This is an appearance I have never seen, nor do I believe it to be of common occurrence. I am well satisfied that in nine tenths of the cases of anchylosis following ulceration of the cartilage, the bands of adhesion by which the anchylosis is effected have been effused from the surface of the denuded bone."

Mr. Blackburn thinks osseous anchylosis much less common than is generally supposed:

"Crowther, whose experience was very considerable, says, 'I have seen but two cases of the knee and one of the elbow anchylosed by ossific union;' and so far as my own observation extends the inference to be drawn from his testimony is confirmed. The fallacy has probably arisen partly from the inspection of pathological museums, for which the specimens of osseous anchylosis are treasured up, while those of ligamentous union are thrown away, and partly from a common though erroneous belief, that soft anchylosis always admits of some degree of motion."

The author has made several judicious observations on the prevention and treatment of anchylosis, but we do not observe any point requiring particular notice. He concludes by relating the remarkable case of anchylosis of the hip-joint, which was remedied by Dr. Barton of Philadelphia, by sawing through the neck of the bone, and allowing an artificial joint to form;* and offers the following comments upon it:

"Before we give our decided approbation to this operation, the important question has to be considered, does it endanger the patient's life? If we look again to the somewhat analogous though more severe operation of excision, we find that it has been almost invariably successful when performed in the elbow-joint, generally on the shoulder-joint, never on the hip, where indeed it has been but once attempted, and rarely on the knee. There are many reasons which the reader will call to mind, why an operation such as that proposed by Dr. Barton should be more successful than that of excision; but I cannot bring myself to believe that even the former can be performed on the lower extremities without considerable danger. The large size and depth of the wound must oftentimes give rise to severe irritative fever or exhausting suppuration. These are risks which the evils of anchylosis do not warrant us in incurring; at least, I should find it difficult to wash from my hands the blood of any patient of mine who died in consequence of an operation for the relief of anchylosed hip or knee."

We observe that Sanson gives a similar though less strongly expressed

* Br. and For. Med. Rev., July, 1838.

opinion against the operation,* nevertheless, the question is one that cannot be decided by authority; and we are not quite sure that these gentlemen in coming to the above conclusion have not leaned too much on the supposed analogy between Dr. Barton's operation and that of excision of the heads of the bones in the lower extremities, which is certainly a much more severe and complicated operation. We may observe in support of our view that the operation has been twice repeated in America with success.†

*Chrondritis.* The article on the inflammation of cartilage (chrondritis,) we have already stated to be by Mr. Wells; it is an excellent memoir. After giving an account of the usual symptoms of ulceration of the cartilages, and remarking on the familiar fact, that in disease of the hip-joint the pain is often felt chiefly at the knee, Mr. Wells observes:

"In the other joints the pain is usually referred to the actual seat of disease, but in addition to this, a tormenting sensation of pain and numbness is felt along the shaft of the humerus when the shoulder-joint is the seat of ulceration In the case of a lady I was lately in the habit of seeing, this symptom had completely led her physician from the seat of the disease; he considered it pathognomonic of angina pectoris. In case of disease of the elbow-joint also, pain and numbness are sometimes felt in the wrist and back of the hand."

Great differences exist in different cases both as regards the rapidity with which the local disease makes progress, and the extent to which the constitution suffers. The author witnessed a case "in which there was surprisingly little disturbance of the functions, though, as was proved by examination of the amputated limb, effusion of pus and lymph had taken place into the joint. There was caries of the ends of the bones, external œdema and suppuration; and permanent flexion of the limb with consecutive luxations." Such cases are however rare, as, usually, hectic and wasting diarrhœa attend such local disorganization. In describing the class of cases which occasionally supervene during the progress of erysipelas, phlebitis, and the puerperal state, 'in which rapid absorption of the articular cartilages takes place with acute inflammation of the synovial membrane, the author mentions having met with the following case:

"I had lately the opportunity of examining the body of a female who died about a fortnight after delivery with symptoms which were ascribed to puerperal peritonitis. There were, however, no traces of abdominal disease. While examining the organs of the pelvis, our attention was directed to the left sacro-iliac synchondrosis, by an appearance of bulging in that situation. We found that the cartilages covering the opposed surfaces of the ilium and sacrum had been completely absorbed, and the bones were consequently denuded. There was a large quantity of pus contained between and around the bones, and their abraded surface was covered with an exudation of recent lymph."

Appearances of this kind are far from uncommon in puerperal cases, as may be seen from our article on puerperal diseases in the present Number.

In the treatment of ulceration of the cartilages, "the first object is to keep the affected joint in a state of the most absolute quietude;" for this purpose a calico bandage, stiffened with starch, albumen, or resin, will

* Dict. de Méd. et Chir. pratiques. Article, Ankylose.

† See Lancet for April 18, 1840, and Br. and For. Med. Rev. for July, 1840.

perhaps be found the best fitted. Local bloodletting is proper where the constitutional powers have not been impaired. In children blisters kept open, but in adults caustics are by far the most effectual means. The moxa which Mr. Wells recommends is made with paper which has been steeped in the liquor plumbi diacetatis, and afterwards dried. This is folded into a narrow strip and rolled up after the manner of a bandage, until it gives a surface about the size of a sixpence; Mr. Wells is against opening the joint where matter has formed, as more likely to lead to dangerous consequences than the leaving it to nature.

*Amputation.* This article is a very long one, and is the joint production of the editor, and of Dr. King, whose former post of house-surgeon to the Hôtel Dieu must have given him abundant opportunities for observation in this department of surgery, as conducted by some of the ablest of the French surgeons. After a brief historical notice of amputation, the author of this portion of the article, Dr. King we presume, proceeds to notice the various injuries and diseases which may render amputation necessary. In speaking of compound fracture as one of these, he observes that some surgeons contend that amputation is hardly ever necessary in compound fractures, others that it is frequently called for.

"Velpeau has recorded some bad cases of compound fracture cured without amputation. Sir A. Cooper's practice abounds with them; indeed, it is to him that we are chiefly indebted for the treatment by which such favorable results have of late been obtained in this department of surgery. Dupuytren's hospital practice was far less favorable in these cases, which I attribute to the frequency with which he was accustomed to renew the dressing, and thereby disturb the limb. It contrasted strongly with that of Sanson who adopted Sir A. Cooper's plan of treatment with much success."

Under the heads of Prognosis, Time for amputating, Place at which amputation is to be performed, and Preparation of the patient, are contained many judicious observations, from which we should willingly make quotations did our space admit of it. In speaking of the apparatus required for amputation, the author gives the following caution, which is well worth attending to; namely, that in order to avoid any serious omissions, the operators should, before the operation, take up each instrument in succession in the order in which it is required. Dupuytren, he observes, never neglected these precautions.

The author strongly advocates the employment of the fingers for compressing the artery, in preference to the use of a tourniquet, whenever a trustworthy assistant is at hand to whom the duty can be committed, and the limb is in such a state as to admit of its adoption.

"Those who have never seen it tried," he observes, "will be agreeably surprised to find with what ease and certainty the passage of the blood may be completely stopped in a large artery by the fingers properly applied, provided the vessel rest upon something solid, as the femoral artery does on the os pubis, and the brachial artery on the upper third of the humerus."

In describing the various modes of performing the circular amputation adopted at different periods, the author very properly insists on the advantages resulting, in the modern mode of performing the operation, from division of the integuments, of the superficial muscles, and of the deep layer, by successive incisions, and urges the necessity of preserving an ample supply of integuments to cover the face of the stump. The following account of Dupuytren's operations exhibits the evils arising from want of attention to this point, and may serve as a useful caution against sacrificing to mere rapidity of execution advantages of higher importance:

"Dupuytren, by a strange and inexplicable fatality, operated like Louis in dividing skin and muscles together. He differed from him in making more incisions of the latter, and in having the skin drawn tight and fixed by the hands of an assistant instead of employing a ligature. His incisions were made with such velocity that the eye could scarcely follow them; and to superficial observers, to those who were mere spectators, his amputations appeared splendid. But in myself, who assisted Dupuytren at these operations, the admiration was mingled with regret. I had to watch and dress the patients, and could not shut my eyes to the results. The stumps were often conical in the wrong sense; sometimes even the bone was salient and afterwards exfoliated. Those who witnessed the operation only went away impressed that Dupuytren's amputations were equal to his other operations, almost all of which were excellent. This was unfortunate, for had it been otherwise, the general opinion of the profession would have been against his plan, and he might have been induced to alter it. His great fault lay in not devoting his first incision to the division and preservation of the integuments. This he either did not see or thought he could counteract by preserving an additional quantity of muscle; for he was aware that his stumps were often faulty, and I have frequently seen him make more than three sections of the muscles, and also dissect up round the bone, whispering as he proceeded, 'Surely I shall preserve soft parts enough here!' as if determined to procure a good stump."

The latter half of the article is devoted to the description of particular amputations, and contains a pretty full account of the various modes of amputations recommended by the most eminent surgeons, whether in this country or on the continent.

In treating of amputation at the hip-joint, the author enumerates about seventeen cases in which the operation has been successfully performed; it has however failed, as might well be supposed, in a considerable number of cases, even when performed by the most eminent surgeons. The relative proportion of successful to unsuccessful cases is not known.

Mr. Abernethy recommended the operation to be performed by the circular method, and this mode is also preferred by Gräfe. It has been carried into effect by Mr. Colles and by Krimer. "The majority of the amputations hitherto performed at the hip-joint have however been executed according to the flap method, or some of its modifications." Of the various modes of performing the flap operation, the authors consider that "those which contain most skin and least muscle are best; as the liability to inflammation and extensive suppuration will be least in flaps so formed. This is one excellence in the mode of proceeding recommended by Mr. Guthrie and Dupuytren."

The woodcuts with which the volume is illustrated are very unequal in quality: those contained in the early numbers, as for instance in the present article, are only moderately well executed diagrams. In the latter numbers we are glad to see a decided improvement.

*Angeioleucitis.* This article is written by M. Velpeau, and is a valuable contribution to the Cyclopædia, whether we look at its intrinsic merits as a practical paper on an important form of disease, or consider it in the light of an attempt to supply, what has been hitherto a desideratum in medical literature, a systematic account of acute inflammation of the lymphatic vessels; and to draw the lines of demarcation between this and some other diseases with which it has frequently been confounded, namely, phlebitis and phlegmonous erysipelas. That M. Velpeau has left nothing more to be desired on these several points is more than we will undertake to assert, but we are quite ready to accept this contribution of his towards a general history of the disease, and to thank him for the able manner in which he has broken ground.

Certain forms of angeioleucitis, especially as affecting the superficial absorbents, have long been familiar to practical men, but we should be at a loss to name any very good account even of these; and the want of anything like an accurate history of the disease in general will justify us in giving a somewhat extended summary of the paper before us.

"The lymphatic system," says M. Velpeau, "is capable of becoming inflamed in different ways and in various degrees, like the other tissues of the body. Here as elsewhere we observe inflammations to arise from direct and indirect causes, from contusions, or solutions of continuity, either of the lymphatic vessels themselves or their ganglions [glands]. But this inflammation nevertheless, at its commencement, most commonly has its source in diseases quite foreign to the organs of which we are now speaking; and it will be readily recognized [acknowledged] that almost all the diseases of the lymphatic system arise in consequence of fluids either produced or vitiated by inflammation being introduced therein by absorption or imbibition, and carried from the periphery [surface] of the body to the centre..... If the inflammation, affecting the canalicular [?] portion of the lymphatic system have hitherto attracted so little attention, the reason is, that being in the majority of cases difficult to be recognized directly, they have been either mistaken for or confounded with the diseases of some other system; that of the veins for example."

One essential difference in the character of these inflammations, according to the author, results from the matter absorbed having undergone changes consequent on exposure to the atmosphere, or having been free from this operation.

The symptoms of angeioleucitis are local and general, the former varying according as the disease affects the superficial or the deep-seated absorbents. Inflammation of the superficial absorbents almost always arises from a wound, inflammation, or suppuration of the skin, some unhealthy change, such as an increase of the inflammation or otherwise of the affected part having preceded it.

"An erythematous blush appears round those wounds or ulcers that had been dressed with deteriorated adhesive plaster or rancid applications, or which had been covered with incrustations; streaks, bands, or patches, varying in colour from a bright or rosy red to a wine or violet hue, soon appear at other points of the diseased region. These bands are tortuous, irregular, and cross each other in such a manner as to form small islets of sound skin, and follow the tracks of the lymphatic vessels. They do not show themselves first on the parts nearest to the wound; those first seen are even sometimes at some distance above it."

Erysipelatous patches appear at first disseminated, but afterwards grouped together into a sort of erysipelas, commencing first round the diseased part, from which the inflammation extends to others; or the patches may appear simultaneously at separate points, connected by the red lines or streaks. The pain felt in these red patches is acrid, resembling that of a part sunburnt; the swelling is at first slight, but extends irregularly in depth and surface, and is developed in a knotted form around the lymphatic vessels and glands rather than taking the course of the cellular tissue properly so called.

"At first the tissues retain a certain degree of suppleness notwithstanding the swelling and redness. At a later period the tension increases, and their feeling [?] is like that of sponge; to the finger they seem as if they were œdematous, and are in reality the seat of infiltration; the tension, however, has not a frank,[?] elastic, and regular character, nor is it the same as we find in the doughiness of acute phlegmon or phlegmonous erysipelas. ...... When the attack occurs first in the deep-seated lymphatic vessels, as sometimes happens in the case of wounds or ulcers that pass under the fasciæ, as also of contusion, inflammation, abscess, or ulcer, the symptoms assume a somewhat different character. It is the pain that first attracts notice."

This pain is described as a fixed one, not radiating in lines from a given point, nor diffused, nor remittent, but fixed in particular spots.

"The tumefaction comes on afterwards, or it may occur almost at the same time and in the same places as the pain. It proceeds and is developed from the centre to the circumference. It is observed under the form of masses of more or less extent, of thick and long cores, but there is no appearance of patches as in the case described already. Its origin is without difficulty recognized to be from beneath the fascia, and the tissues are less dense in proportion as they are nearer to the skin, which retains for a long time suppleness and a certain degree of mobility."

The redness is not manifest until later, and is less superficial than in the former variety. The skin is stretched, and has a whitish or pale rose rather than a red colour. Generally speaking, it is the deep lymphatic ganglions that first swell and become painful. "It would however be wrong," observes the author, "to suppose that the two plans [?] of the lymphatics may be thus long affected independently of each other. More frequently, on the contrary, the affection of the superficial lymphatics is communicated to the deep-seated vessels, and vice versâ."

*General symptoms.* These commence sometimes with mere horripilation, at others with irregular rigors alternating with great heat and dryness of skin. "The pulse, always frequent, is at one time strong and full, as in an inflammatory fever, at another small and irregular, as in low fever." Thirst, anxiety at the precordia, nausea or vomiting, are often present. Watchfulness and agitation, with confusion going on often in the course of some days to delirium, denote the affection of the nervous system. "A part or the whole of the above symptoms may be present before the local symptoms become developed." The tongue, at first soft and covered with a grayish or yellow fur, becomes dry and rasplike, and finally cracked as in typhus fever; the gums and mouth are fuliginous.

The symptoms above described, the author thinks, are evidently due in part to the inflammatory process going on, and in part to the infection of the blood from matters absorbed, a mode of accounting for them which appears, to say the least, reasonable.

Angeioleucitis may terminate in *resolution*, especially when the disease is limited to the superficial absorbents, but very frequently suppuration takes place when the red patches are numerous and run into each other, and when painful cores of some thickness are felt beneath them. The pus is either infiltrated along the course of the red streaks or collected in abscesses chiefly below the red patches: fluctuation is not felt till a late period: one abscess is generally a precursor of others; the author once saw twenty-five in the same individual. Though the pus is thin, the secretion soon ceases and the parts heal.

"The termination by *induration* without suppuration is rather uncommon, and occurs almost exclusively in chronic angeioleucitis." When it occurs "the lymph which remains in the state of infiltration in the meshes of the cellular tissue is but slightly altered, and sufficiently concrescible to have a constant tendency to combine with the tissues which it has swollen or hypertrophied, and which it transforms into lardaceous layers and masses.

"Angeioleucitis may terminate in death, either at the time when the inflammatory reaction is at its height, or towards the end, when the suppuration is prolonged. It may be apprehended in the first case when high delirium, a dry mouth and nausea, accompany an extensive inflammation, which continues unabated till the eighth or tenth day. It may also be dreaded in the case where the inflammation is extensive, deep and severe, more especially in an individual of a broken constitution or advanced age, and when the suppuration threatens from the first to be vast and abundant. At a later period the fatal termination is brought on by the recurrence of the abscesses, through the exhaustion arising from the abundance of the purulent secretion, from the occurrence of fresh attacks in the viscera, or from effusion into the serous cavities, and vitiation of the blood by its mixture with the pus or absorbed substances."

The diseases with which, according to M. Velpeau, angeioleucitis is most likely to be confounded are phlebitis, erysipelas, neuritis or neuralgia, and phlegmon. The author has entered at some length into the diagnosis between this disease and phlebitis, and we should willingly transfer his observations to our pages but that they are too extended to allow of this. The principal points are, that in phlebitis the red bands are fewer, deeper, and less frequently crossed, and more like hard cords. The red patches are less constant and fewer. Suppuration is more rapid, and the pus looks as if mixed with decomposed blood; and when several abscesses occur, they are developed in the course of the venous trunks, and not in different regions of the limb. Tumefaction is less, and the skin neither tense nor shining, but thickened or inflamed. The lymphatic glands are unaffected. The general symptoms in phlebitis have less of the inflammatory and more of the adynamic character, stupor and prostration sooner come on, and death more certainly ensues when the general symptoms are developed than in angeioleucitis.

"As a local inflammation the former is very easy, the latter very difficult to check. As a general affection the case will be exactly the reverse of this. .... It must, however, be admitted that these two forms of inflammation exhibit a close affinity in regard to their causes, their mode of production, and their deadliness. It would therefore be improper to attempt to isolate them by means of perfectly defined signs and limits. Their tendency to unite both at the commencement as well as through their course, the symptoms that are common to both, and the difference as regards the dangers incident to them are quite enough to challenge all the attention that the practitioner can bestow on the distinctive characters of these two diseases."

With regard to some of the other affections with which angeioleucitis is sometimes confounded, such as neuralgia or common erysipelas, the diagnosis is more easy, and we think it hardly necessary to quote any of M. Velpeau's observations on this part of the subject. In phlegmonous erysipelas the differences are perhaps less strongly marked, but even here the gradual progress of the disease, involving all parts through which it spreads, instead of the appearance of scattered patches or indurated cores, the rapid formation of purulent matter, the absence of swelling in the lymphatic glands, and the late appearance of serious general symptoms are sufficient to distinguish the two diseases. Not unfrequently, however, the diseases are complicated with each other.

"A most remarkable instance of this," observes M. Velpeau, "occurred in my own wards in the months of March, April, and May, 1837: almost all the patients that had been affected with angeioleucitis were soon after attacked with erysipelas strictly so called, or with phlegmonous erysipelas, and in like manner those who were first attacked with erysipelas soon presented it complicated with angeioleucitis."

The pathological alterations resulting from inflammation of the lymphatics consist of changes in the vessels themselves, in the tissues around them, or in distant organs. Even when surrounded by broken-down and suppurating tissues, the lymphatics are often found still permeable, with thickened coats and their inner surface slightly tomentous, especially where vessels meet or valves are numerous. The surrounding cellular tissue is in some places healthy, in others more or less disorganized, infiltrated, hardened, or broken down. The muscles and nerves are little altered. The blood, when the disease is of some standing, is generally very fluid. Pus has not been found in it. In the parenchymatous viscera abscesses are rarely found, and when present are small. Sero-purulent effusions are frequently observed in the pleuræ, peritoneum, and joints. "On the whole," observes the author, "the internal lesions found after death are often very disproportionate to the symptoms observed during life." The plan of *treatment* found most successful by M. Velpeau is as follows:

"If any wound exist, whether it be recent or of long standing, it is covered with a thick poultice: if there be any arterial reaction, recourse is had to general bleeding; the quantity abstracted to be somewhat considerable, to be followed up by the use of a tepid bath for an hour; twenty or thirty leeches are applied round the wound when it appears red and swollen. After these means compression with a roller will be of use, its action will be promoted by wetting it several times during the day with some resolvent fluid. Cold water perhaps would answer very well in this way. If compression is not followed by the desired effect, mercurial inunction is indicated. The plan I pursue is to have two drachms rubbed in three times in twenty-four hours over the entire extent of and a little beyond the painful region........... As soon as any fluctuation can be detected, no matter how obscure at any point, the bistoury must be made use of at once, all these abscesses requiring to be largely and promptly opened. At this stage recourse may again be had to poultices over the suppurating parts, and to compression if the form of the part will allow of it. When resolution does not take place, and the suppuration is tardy, it will be right to employ blisters. They may be applied in succession or simultaneously over the points where the inflammation was most intense, and which are still the most engorged. The larger the blisters the more certain their good effects, acting either as the best maturatives or the best resolvents that I know of. In many such cases I can speak decidedly as to their good effects, having succeeded in a little time by means of them either in dispersing or producing suppuration in masses which seemed to be unalterably indurated. When there is delirium or great agitation I also employ them from the beginning, arranging them in such a manner as to cover over the whole of the diseased with some of the sound parts. They are removed in twenty-four hours with the epiderm, and the exposed parts are powdered with camphor to protect the urinary passages."

In another place M. Velpeau says, "I am not afraid, for example, to envelope the whole limb, from the shoulder to the finger, or from the groin to the knee, or from the knee to the toes. The general and local symptoms are by this treatment arrested so rapidly, that in a week or two a revolution which could scarcely have been expected is effected. This plan of treatment may justly be considered as heroic."

As regards chronic angeioleucitis the appearances presented are so different from those exhibited in the acute disease, that it is everywhere described under other appellations. M. Alard has shown that it forms an important element in elephantiasis, as it does also in other chronic diseases of the skin.

In the latter part of this volume there are several contributions from the hand of Mr. Spencer Wells. The article Bronchotomy consists in the main of an essay for which he obtained the prize of the Dublin Medico-Chirurgical Society for 1839, preceded by a historical review of the subject from the earliest ages of surgery to our own times. The article is a very good one, and we congratulate Dr. Costello on having added so able a working member to his list.

The articles on Midwifery in the eighth number, namely, Crotchet, Detruncation, and Deliverance, are by the same hand. The two former are brief and hardly call for comment In the latter we see little to object to except the title, one of those new importations with which Dr. Costello has frenchified his work, and very unnecessarily, since there is often nothing, as in the present case, to recommend the adopted name. *Délivre* is a term in common use for the placenta in France, and *delivrance* is an unobjectionable derivative, but to the English ear deliverance conveys no meaning except one likely to be confounded with delivery. Under the head of Placenta might very well have been included all that is here given, indeed some of the discussions would have been much more appropriately entered on under that title than under deliverance. Had we not entered so largely in our last Number into the discussion of obstetrical questions, we should have been glad to have enriched our pages with some extracts from these able papers of Mr. Wells.

The articles *Balanitis*, *Bubo*, and *Condyloma* are by Mr. H. J. Johnson. The latter "is substantially a reprint of a paper which appeared in the Medico-Chirurgical Review for July, 1834, with such alterations and additions as further experience and the publications of other gentlemen have given birth to."

Mr. Johnson arranges condylomata under three heads: the first he terms the *simple condylomatous excrescence*, as it occurs independently of venereal taint, and is merely the result of irritating secretions; the second and third kinds he calls *non-ulcerated venereal condyloma*, and the *ulcerated venereal condyloma.* "The second and third varieties," says Mr. Johnson, "are probably rather grades of one than possessed of specific differences. And yet they do differ."

The second form of condyloma he describes as a flat, superficial, nearly circular deposit in the cutis. It is soft and smooth on its surface, is evidently vascular, generally of a pinkish sometimes of a bluish tint, according to the locality and the vigour of the circulation; it varies in thickness from that of a sixpenny to nearly that of a penny-piece; in circumference it seldom exceeds the former and is often little larger than a split pea.

The third form is distinguished by the fact of ulceration having taken place on the surface of the deposit. "On some the ulceration is very superficial, the appearance being that of a mucous membrane or a fine moistened cuticle; on others there is distinct flat ulceration; and on others the ulceration may be rather cupped. The tint is generally red; seldom, save in the cupped ulcerations, yellow. The base or substratum of all is firm, for the obvious reason that each is a condylomatous deposit."

These forms of condyloma are in either sex attended with a discharge like that of chronic gonorrhœa. "I presume," says Mr. Johnson, "that in many if not in most instances the discharge and the condylomata stand in the relation of cause and effect to each other. But once produced, the condylomatous sores undoubtedly have the property of giving rise to fresh ones both in the same and other individuals." The facts which have been mentioned prove the first position. The second is established by artificial inoculation as well as by experiments of a less premeditated character. Three cases are related, in the first of which a gentleman acquired what seemed a common syphilitic sore from a female who was found on examination to have the labia and inside of the thighs studded with ulcerated condylomata, with a vaginal discharge, but no other sores. Both patients got well under the use of mercury. In the two latter, condylomatous sores appeared on the penis and scrotum after ulcers which had assumed a phagedenic aspect under the use of mercury. These forms of condyloma, especially the third, are followed, says Mr. Johnson, by unequivocal secondary affections, and he therefore considers them "entitled to rank amongst the forms of syphilis." He enumerates bubo, ulceration of the throat, affections of the tongue and lips, and various cutaneous affections, as sequelæ of condylomata.

"The condyloma attends chronic discharge, is probably generated by it, and forms perhaps the link between those affections of the mucous membrane of the vagina and urethra which seldom occasion secondary symptoms, and the sores which are so prone to do so. The study of condyloma seems to me to derive a large share of interest from this consideration, for if it be correct, we find in condyloma a sufficiently powerful machinery for the constant production of specific sores, and an argument of no mean force in the hands of those who contend for the extreme antiquity of syphilis."

Mr. Johnson's communications are all practical and sound, and therefore valuable.

The articles upon Diseases of the Eye are by Mr. Tyrrell, Mr. Middlemore, Dr. A. Watson, and Mr. T. W. Jones. They all appear to be well executed; but we must postpone any notice of these until some future period.

Several other papers of value would have claimed attention from us at the present time had we not already noticed them in former Numbers of our Journal at the time of their publication: amongst these are Mr. Tyrrell's paper on Amaurosis, Dr. Wardrop's on Aneurism, and Dr. Walshe's on Cancer. An extended notice of Dr. Willis's work on Urinary Diseases, and of Dr. Wardrop on Bloodletting at the time they appeared will also justify our passing unnoticed at present their contributions on Calculus and Venesection. We cannot, however, conclude this article without noticing, at some length, an excellent paper by Dr. Walshe on a subject which has been but little investigated in this country, and, we believe, is but ill understood.

*Cephalhæmatoma.* We believe we are correct in stating that the profession is, in the article before us, indebted to Dr. Walshe for the first systematic and complete essay in the English language, upon this very peculiar and interesting affection. We trust that the circulation of his performance will put an end to the curious confusion existing in the minds as well as the writings of obstetricians respecting the nature of the affection; that this will at length cease to be considered identical with or a mere modification of the common scalp-swelling or sero-sanguineous œdema of new-born infants. We may take credit to ourselves also for having in previous Numbers of this Journal laboured in this field, a fact which will be the apology for our scarcely entering into the subject at present.

Dr. Walshe recognizes three species of the affection according to the precise situation of the effused blood: the *extra-cranial*, the *intra-cranial*, and the *interstitial*. The former species is divided into two sub-species, the *sub-pericranial* and the *extra-pericranial*, terms which explain themselves. The interstitial species Dr. Walshe admits rather interrogatively.

The writer states that one of the objects proposed in his essay is to "exhibit, in the present diversity of opinion on some important points, the ample field still left for accurate enquiry." We shall indulge in one extract sufficiently proving that Dr. Walshe has not failed in his purpose:

"The contradictory opinions held on the anatomical condition of the parts in this affection leads us to expect anything but harmony on the subject of its causes and mechanism. Numerous observers contend that it is caused by violence directly applied to the part during protracted labour, either from impaction (Feiler), or pressure against the sides of the pelvis (C. C. Klein); or from contusion with the forceps, &c. (F. B. Osiander.) But these practitioners, and others who have adopted very similar notions, have doubtless confounded the affection, which we now describe, with common œdematous scalp-swellings; for accurate observation has proved that it occurs most frequently after easy labour, during which the head has not undergone any notable pressure. Not a single one of Naegele's cases occurred after laborious parturition; Meissner, Hüter, and Valleix have observed it in infants extracted by the feet; it has been found in the second born of twins presenting the breech; and we have seen how frequently its existence has been ascertained before or immediately after the rupture of the membranes. Hence the opinion referring the affection to severe pressure or contusion is untenable. Michaelis ascribes the effusion to the exposure of the diploe resulting from the carious destruction of the external table; of this opinion we have already seen the error. Naegele vaguely refers the rupture of vessels to abnormal development of the bone; Meissner and Neumann to displacement or riding of the bones of the scalp during labour; Brandau to a congenital relaxed state of the vessels of the head; Stein fancies the disease produced by an abnormal disposition of the vessels; Siebold by a state of the vascular system of the bone analogous to the aneurism by anastomosis of the soft parts. All these notions are either clearly shown to be incorrect by the previous description, or at the best insignificant and hypothetical. Busch having observed a large blood-tumour on the occiput of a child delivered with the forceps, which communicated with the sinuses, deduced a general theory of the disease from this example of a totally different affection. Dzondi believes the proximate cause of the affection to consist in imperfect development of a part of the parietal bone, the diploe of which is *at the time of birth uncovered by an external table.* The delicate vessels of the diploe are burst, he supposes, by the forcible rush of blood to the head, consequent on the action of the uterus or of pressure against the sides of the pelvis. The imperfect development of the bone in the affected part he attributes to the continuous pressure of the promontory of the sacrum on the head, and especially on either parietal bone. M. Paul Dubois considers that from the extreme facility with which blood may be expressed from the infant cranium after the separation of its investing membranes, such separation, if it occur during life, must lead to the formation of cephalhæmatoma, and hence that the causes of the former are the true problem. These, he maintains, may be of various kinds, either, for example, unnaturally lax union of the pericranium and bone, violence down to the head, or disease of the osseous tissue itself."

Dr. Walshe then explains the doctrine which has recently been maintained by M. Valleix; of this we have elsewhere stated the substance.* The author states the opinion which he has himself been led to form upon the cause of the affection in the following terms:

"On the whole it appears that the effusion mediately depends on imperfect development of the external table of the bones, and lax adhesion of the pericranium; immediately upon any causes affecting a separation between the latter and the bony surface of these, circular constriction of the cervix uteri is probably the most frequent, but certainly not the sole, as indeed M. Valleix himself allows may be the fact. Respecting the state of the bone itself and the influence of this on the effusion, it will be observed that there is the evidence of three authors in favour of the absence of the external table; that of Michaelis, who conceives it due to local caries; of Dzondi, who ascribes it to local abnormal suspension of development from pressure; of Valleix, who maintains such imperfection the natural condition at this period of existence. That it is wanting in, at least, some cases of cephalhæmatoma is more than probable, but this may be accounted for in various ways; it is not impossible that in cases preexisting to birth the collection of blood may have really formed in the diploe and destroyed the ill-formed outer table by pressure; were this the fact, subpericranial would be only an advanced stage of interstitial cephalhæmatoma."

The following paragraph on the diagnosis of the affection is valuable:

"Among the affections for which this form of cephalhæmatoma is liable to be mistaken is *congenital encephalocele.* The points of distinction between them are nevertheless, as may be gathered from the subjoined enumeration, numerous and clearly defined:

| " *Subpericranial Cephalhæmatoma.* | *Hernia of the Brain.* |
|---|---|
| Is the seat of fluctuation. | There is no fluctuation, unless water be contained in the hernia. |
| Is perfectly indolent. | Is occasionally the seat of pain, especially under pressure. |
| Pressure produces no cerebral symptoms. | When large, cerebral symptoms frequently arise spontaneously; these are greatly increased by pressure. |
| Nor does it change the size of the swelling. | Pressure diminishes its bulk, which it reacquires when the pressure is removed. |
| The investing skin and hair are in the natural state. | The skin is thinner than natural, and without hair. |
| Is never seated on the sutures. | In the vast majority of cases is seated on the sutures or fontanels. |
| Is tense and hard. | Is elastic and soft. |
| Pulsation is extremely rare. | Pulsation is an ordinary symptom. |
| Is uninfluenced by the respiratory movements. | Swells during expiration, and sinks during inspiration. |
| Apparent perforation, caused by the elevated bony ridge. | Real opening in the skull, either between the sutures or through the substance of the bones. |
| Is never pedunculated. | Inclines to the pedunculated shape, which is often very marked. |

* Vide Br. and For. Med. Rev., vol. VII. p. 89. Jan. 1839.

"It seems hardly possible to confound this affection with ordinary *hydrocephalus;* a *subfascial aqueous cyst*, spoken of in Zeller's thesis, might be distinguished by the absence of the bony elevated rim. *Erectile tumours* of the scalp are soft, spongy, easily depressible, lessened in size by pressure, recovering their former dimensions by successive influxes of blood, expand when the infant cries or coughs, are of a bluish-violet tint, have no prominent elevated border, and are not the seat of fluctuation;—the characters of cephalhæmatoma are in all respects the reverse. *Encysted tumours of the scalp* are distinguishable by their being moveable, and by the absence of the elevated margin. *Perforating cancer of the meninges* is reducible, is not surrounded with a raised rim, and is otherwise specially characterized. *Collections of pus* under the pericranium, which may possess almost every external character of cephalhæmatoma, even to the presence of an elevated and indurated margin, M. Valleix presumes might be distinguished therefrom by the slow and gradual formation of the tumour, the pain in the part (?), and tenderness on pressure, as well as inferior degree of hardness of the surrounding prominence, which, it appears, contains no osseous matter, and is composed of hardened cellular tissue. *Common scalp swellings* are characterized by being almost invariably the result of difficult labour, by being seated on the part descending first into the cavity of the pelvis, by being always single, indistinctly circumscribed, of deep livid colour, non-fluctuating, pitting on pressure, rarely surrounded by an annular prominence, and disappearing in from twelve to forty-eight hours. The distinctive marks of extra-pericranial cephalhæmatoma will be presently stated. M. P. Dubois once met a combination of œdematous scalp swelling, extra and sub-pericranial cephalhæmatoma; under such circumstances an accurate diagnosis must be next to impossible."

We must refer to the original for a very full investigation of the therapeutical questions relating to the disease; but we cannot conclude without expressing our high opinion of the value of this communication, and of the clear and precise style in which it is written.

Several other papers in this volume are well deserving of attention, but from about 100 articles which it contains it was clearly necessary that a selection should be made; and this we have endeavoured to do in such a manner as to give the reader a fair estimate of the merits of the work. These merits are very considerable; and we feel that we may with a clear conscience, not only commend the first volume of the Cyclopædia of Surgery to a place on the shelves of all our readers, but state that the library of no modern surgeon can be complete without it.

The reader of taste will not have failed to notice the prosy and ungainly title of the work prefixed to this article. He will also have observed, in some of the extracts we have given, specimens of the outlandish diction wherewith it abounds, and which the learned Editor is pleased to inflict upon his readers as English. We wish we could say that these are the only editorial blemishes with which the work is chargeable. Still, as it is not the first time we have heard of gallant armies gaining victories in despite of their generals; and as we think most highly of the skill and courage of the Cyclopædian troops, and of the spirit and liberality of the central government in Paternoster Row, we look forward with confidence to the successful termination of the present surgical campaign.

